# Causes of Hospitalization for Systemic Lupus Erythematosus in Saudi Arabia Compared With the Global Setting: A Retrospective Single-center Observational Study

**DOI:** 10.7759/cureus.18858

**Published:** 2021-10-18

**Authors:** Noor Alhassan, Talal Almetri, Shada Abualsoud, Alaa Malhis, Khaled Al-Qahtani, Abduallah Alwazna, Nourhan Salloum, Bandar Zaeri, Asmaa Hegazy, Sara Mohamed, Yara Bashawri, Nayef Al Ghanim

**Affiliations:** 1 Internal Medicine Department, King Faisal Specialist Hospital and Research Centre, Riyadh, SAU; 2 Internal Medicine Department, Dalhousie Medical School, Halifax, CAN; 3 School of Medicine, Royal College of Surgeons, Dublin, IRL; 4 Internal Medicine Department, King Saud Medical City, Riyadh, SAU

**Keywords:** intensive care unit, infection, lupus flare, hospitalization, systemic lupus erythematosus

## Abstract

Background: This study sought to evaluate the main causes of hospitalization of patients with systemic lupus erythematosus (SLE) in a tertiary health center in Saudi Arabia.

Methods: A retrospective observational study was performed for all the SLE patients admitted to King Saud Medical City between 2016 and 2019. The primary reason for hospitalization was determined by the primary physician caring for the patient at the time of admission.

Results: Of the 98 hospitalizations for SLE, 49% of patients were admitted from the emergency department (ED) and 51% from the rheumatology clinic. The most common reason for hospitalization was lupus flare (68.4%) followed by infection (20.4%). The lupus flare patients commonly presented with *musculoskeletal* (MSK)symptoms (34.6%), renal manifestations (25.5%), and skin rash (24.5%), whereas patients admitted with infection were commonly diagnosed with community-acquired pneumonia (12.2%). Other hospitalization causes were obstetric complications, adverse drug reactions, and thrombosis. Intensive care unit (ICU) admission was necessary for 7% of patients due to acute respiratory distress syndrome (ARDS) and pulmonary hemorrhage (28.6%) or other reasons (14.1%), such as pleural effusion, cardiac tamponade*, and thrombotic thrombocytopenic purpura *(TTP)*.*

Conclusions: The two most common reasons for SLE hospitalization were lupus flare and infection. Lupus flare was mainly due to MSK, renal, and dermatologic manifestations. The most common infection leading to hospitalization was community-acquired pneumonia, and ICU admission was mainly due to ARDS and pulmonary hemorrhage.

## Introduction

Systemic lupus erythematosus (SLE) is an autoimmune disease of unknown etiology with multiorgan involvement leading to different clinical manifestations [1,2]. Among hospitalized patients, SLE has high morbidity and mortality rates [3,4]. The causes of hospitalizations of SLE patients have been investigated and multiple reasons identified [5,6], of which lupus flare and infection are the two most common [5-7].

Limited data are available about causes of hospitalizations and outcomes for SLE patients in Saudi Arabia. A 2018 study by Somaily et al. investigated this population at Aseer Central Hospital (Aseer, Saudi Arabia) and found that lupus nephritis (33.9%) followed by infection (16.3%) were the main causes [8]. A 2004 study by Alzeer et al. found 29.2% mortality for SLE patients admitted to the intensive care unit (ICU) [9]. Other published studies did not investigate the causes, morbidity, and mortality of hospitalized SLE patients [8,10-12].

It is essential to identify the main reasons for the hospitalization of SLE patients to enact preventive measures that can decrease the incidence of hospitalization, morbidity, and mortality for this population in the future. Therefore, this study investigated the main causes of hospitalization for SLE patients at the authors’ practice center in a tertiary care center in Riyadh, Saudi Arabia to identify any major differences between groups and to compare findings with previous studies internationally.

## Materials and methods

A retrospective observational study was performed for all SLE patients admitted to King Saud Medical City between January 1, 2016, and December 31, 2019. Patients were identified through the electronic medical record discharge summaries using the International Classification of Diseases, tenth revision, Clinical Modification code M32.9. The patients were included if they met the SLE diagnosis based on the American College of Rheumatology classification/Systemic Lupus International Collaborating Clinics Criteria 2012. Exclusion criteria were age <18 years at the time of hospital admission or missing information regarding the primary cause for admission. The study was approved by the Institutional Review Board. Informed consent by the patients were waived as patients’ personal information were concealed.

The primary reason for admission was determined by the primary physician caring for the patient at the time of admission. The chart was reviewed by a consultant rheumatologist if no clear primary reason for admission was documented. Infection was determined by the presence of clinical signs and symptoms that are suggestive of a process involving a particular organ. For example. Pneumonia was diagnosed by the presence of cough, sputum production, worsening dyspnea, fever, and radiographic features that do suggest an inflammatory process. SLE flare was determined based on their system of involvement, and the flare was diagnosed after ruling out the infection based on the primary rheumatologist's assessment.

Data were collected over three months and included the following information from electronic and paper medical records: demographic data (age, sex, and nationality), time of SLE diagnosis, medications, primary reason for admission, type of admission (through emergency department [ED] or rheumatology clinic), duration of hospitalization, ICU admissions, and mortality.

Previous reports have identified lupus flare and infection as the two most common reasons for hospitalization [1,6-8]. Therefore, the present study compared the patient admissions for lupus flare with those for infection to identify any major differences between the two groups. The study was approved by King Saud Medical City Institutional Review Board (H1RI-07-Feb21-02). The patient's privacy was protected by keeping the data sealed with a password known only by the investigators.

Since SLE flare can overlap with the infection. We did rely on the most responsible physician's judgment in classifying either the patient had had an SLE flare or an infection.

Statistical analysis

Demographic and clinical characteristics of study patients are reported as mean ± standard deviation (SD) or median (minimum, maximum) for continuous variables and numbers (percentages) for categorical variables. To examine differences in the categorical variables between the lupus flare and infection groups, the chi-square test was used, and the Mann-Whitney U test was used for continuous variables. All statistical analyses were performed using the statistical software SPSS 25.0 (SPSS Inc., Chicago, IL, USA). A p-value of <0.05 was considered statistically significant.

## Results

A total of 98 hospital admissions were done for 45 patients with SLE between 2016 and 2019. Some of the 45 patients were admitted more than once. The male-to-female ratio was 1:5.5, and the mean age was 35.78 years (Table 1). Most patients (91.84%) were of Saudi nationality. Admissions were either from the ED (49%) or the rheumatology clinic (51%). The mean duration of hospitalization was 13.16 days. Admission to the ICU was necessary for 7% of patients because of acute respiratory distress syndrome (ARDS) and pulmonary hemorrhage (28.6%) or other reasons (14.1%), such as pleural effusion, cardiac tamponade, and thrombotic thrombocytopenic purpura (TTP). The mean duration of hospitalization among ICU patients was 21.86 days. Among the SLE patients hospitalized secondary to ARDS, one death was reported.

**Table 1:** Main demographic characteristics

Demographic data		No (%)
Gender	Male	15 (15.31%)
	Female	83 (84.69%)
Ethnicity	African	1 (1.02%)
	Filipino	3 (3.06%)
	Indonesian	1 (1.02%)
	Pakistani	1 (1.02%)
	Saudi	90 (91.84%)
	Sudanese	2 (2.04%)

Clinical manifestations at the time of diagnosis are presented in Table 2. The most common clinical manifestations were arthritis (57.14%), proteinuria (51.02%), and serositis (29.59%). Also, 15% of patients were diagnosed with antiphospholipid syndrome (APS).

**Table 2:** Clinical manifestations at the time of diagnosis CNS, central nervous system; SLE, systemic lupus erythematosus

Clinical manifestations	n	%
Oral/nasal ulcer	14	14.29%
Hair loss	12	12.24%
Malar rash	26	26.53%
Other skin rashes	22	22.45%
Discoid lupus	5	5.10%
Photosensitivity	6	6.12%
CNS	25	25.51%
Serositis	29	29.6%
Proteinuria	50	51.02%
Hematuria	5	5.10%
Arthritis	56	57.14%
Thrombosis	14	14.3%
Obstetric antiphospholipid syndrome	1	1.0%

The two most common reasons for hospitalization were lupus flare (68.4%) and infection (20.4%). Patients admitted with lupus flare commonly presented with musculoskeletal (MSK) symptoms which include joints pain, stiffness, arthralgia, and limited range of motion (34.6%), renal manifestations (25.5%), skin rash (24.5%), and serositis (16.3%). Patients admitted with infection were commonly diagnosed with community-acquired pneumonia (12.2%). However, it was noted cutaneous manifestations were present along with other end-organ damage involvement and were not the only reason for admission. Other causes of admissions included obstetric complications, adverse drug reactions, and thrombosis (Table 3). Medications taken at the time of admission are presented in Table 4.

**Table 3:** Causes for hospitalization CNS, central nervous system; MSK, musculoskeletal; UTI, urinary tract infection

Reason for hospitalization		n	%
SLE Flare	Renal	25	25.5%
	CNS	15	15.3%
	Serositis	16	16.3%
	Pulmonary hemorrhage	2	2.0%
	Hematological	7	7.1%
Infection	Pneumonia	12	12.2%
	UTI	1	1.0%
	Gastroenteritis	2	2.0%
	CNS	4	4.1%
	Other	2	2.0%
Drug-related	Medication	2	2.0%
Other	Abdominal wall hematoma	1	1.0%
	Pregnancy complication	3	3.1%
	Surgery	2	1.0%
	Thrombosis	2	2.0%
	Uncontrolled hypertension	1	1.0%

**Table 4:** Medications taken by the patients at the time of admission Ca, calcium

Medications	n	%
Hydroxychloroquine	85	86.7%
Methotrexate	19	19.39%
Azathioprine	13	13.27%
Mycophenolate	12	12.24%
Prednisolone (dose)	82	83.7%
Rituximab	3	3.06%
Belimumab	3	3.06%
Cyclophosphamide	8	8.16%

Laboratory results revealed positive anti-double-stranded DNA in 46 out of 83 tests performed (55.4%), normal C4 (mean 0.16 ± 0.14) and low C3 (mean 0.77 ± 0.41). White blood cell count and lymphocytes count were normal (mean 6.62 ± 4.55 and 1.89 ± 5.90, respectively; Table 5).

**Table 5:** Laboratory results at the time of hospitalization ANA, antinuclear antibodies; Anti-DNA, anti-double strand antibodies; ACLA, anti-cardiolipin antibodies; AB2GPA, anti-beta 2 glycoprotein antibodies; RF, rheumatoid factor; Anti-U1RNP, Anti-U1-ribonucleoprotein; WBC, white blood cells; C3, complement 3; C4, complement 4

Laboratory results	n	%	Mean ± SD
ANA	64	100.0%	
Anti-DNA	46	55.4%	
ACLA	28	43.8%	
AB2GPA	7	30.4%	
Anti-smith	22	46.8%	
RF	21	55.3%	
Anti-U1RNP	11	27.5%	
Anti-Ro/SSA	18	47.4%	
Anti-La/SSB	16	43.2%	
WBC	94		6.62 ± 4.55
Lymphocytes absolute count	90		1.89 ± 5.90
Neutrophils absolute count	89		5.30 ± 6.26
Hemoglobin	95		9.70 ± 1.95
C3	68		0.77 ± 0.41
C4	69		0.16 ± 0.14

Patients with lupus flare (68.4%) and those with infection (20.4%) were compared to identify any major differences between the two groups. A lower mean age for lupus flare versus infection was shown (31.66 ± 14.42 vs. 45.85 ± 12.98, respectively). Most patients in both groups were female (82% lupus flare vs. 90% infection). Fewer admissions through the ED were made for patients with lupus flare (54%) versus infection (70%). No significant difference in the mean duration of hospitalization was observed (13.97 ± 16.52 for lupus flare vs. 11.3 ± 13.62 in infection). Most admissions to the ICU were made for patients with lupus flare (86%) versus infection (14%).

Patients admitted with lupus flare were more likely to have oral and nasal ulcers, hair loss, malar rash, discoid lupus, and other types of skin rash (Table 6); however, these findings were not statistically significant. Comparison of drugs between the two groups showed no statistically significant difference, although a higher rate of prednisolone intake among patients with lupus flare (94%) versus infection (70%) was noted (p = 0.004). There was no difference between the mean dose of prednisolone administered to patients with lupus flare and that given to patients with infection (mean 61.5 ± 211.7 mg and 89.5 ± 262.4 mg, respectively, p = 0.69).

**Table 6:** Clinical manifestations and medications for patients with flare versus infection CNS, central nervous system; Ca, calcium

Clinical manifestations and medications	Lupus flare		Infection		p-value
	n	%	n	%	
Oral/nasal ulcer	13	19.40%	1	5.00%	0.17
Hair loss	11	16.42%	1	5.00%	0.28
Malar rash	23	34.33%	3	15.00%	0.10
Other skin rashes	20	29.85%	2	10.00%	0.07
Discoid lupus	5	7.46%	0	0.00%	0.59
Photosensitivity	5	7.46%	1	5.00%	0.99
CNS	18	26.87%	4	20.00%	0.54
Serositis	17	25.37%	8	40.00%	0.22
Proteinuria	37	55.22%	10	50.00%	0.68
Hematuria	4	5.97%	1	5.00%	0.99
Arthritis	40	59.70%	11	55.00%	0.71
Thrombosis	9	13.43%	3	15.00%	0.99
Obstetric	1	1.49%	0	0.00%	0.99
Hydroxychloroquine	64	95.52%	16	80.00%	0.02
Methotrexate	12	17.91%	6	30.00%	0.35
Azathioprine	9	13.43%	3	15.00%	0.99
Mycophenolate	10	14.93%	1	5.00%	0.45
Prednisolone	63	94.03%	14	70.00%	0.004
Cyclophosphamide	7	10.45%	1	5.00%	0.68
Rituximab	2	2.99%	1	5.00%	0.54
Belimumab	3	4.48%	0	0.00%	0.99

Laboratory results revealed that there were no statistically significant intergroup differences, except for the fact that patients with infection presented with a lower lymphocyte count (0.99 ± 0.61, p = 0.01) than those with a lupus flare (2.22 ± 7.02, p-= 0.01; Table 7). There was also no difference in the rate of positive anti-dsDNA between the two groups (60.66% for lupus flare vs 43.75% for infection, p = 0.22).

**Table 7:** Laboratory results of SLE patients hospitalized with lupus flare versus infection WBC, white blood cells; C3, complement 3; C4, complement 4

Lab results	Lupus flare	Infection	p-value
	Mean ± SD	Mean ± SD	
WBC	6.63 ± 4.45	6.77 ± 5.72	0.49
Lymphocytes	2.22 ± 7.02	0.99 ± 0.61	0.01
Neutrophils	5.5 ± 6.87	5.37 ± 5.32	0.80
Hemoglobin	9.97 ± 1.8	8.83 ± 2.34	0.02
C3	0.73 ± 0.39	0.82 ± 0.56	0.85
C4	0.15 ± 0.15	0.19 ± 0.14	0.46

## Discussion

This study demonstrated that the two main causes of hospitalization among SLE patients were lupus flare and infection. These results are compatible with a study by Liang et al. in the Chinese population for which lupus flare (50.6%) and infection (36.1%) were the most common reasons for hospitalization [13]. In addition, da Rosa et al. showed a similar result among SLE patients in Catalonia, Spain, hospitalized with lupus flare (40.2%) and infection (19.2%) [14]. However, among Israeli SLE patients, lupus flare (20.3%) and pregnancy/labor (18.9%) were the two most common reasons for hospitalization [15], with infection as the third most common cause (13.8%). The frequency of hospitalization for SLE patients secondary to lupus flare (17.5%) and infection (16.2%) appears to be lower among the Canadian population; this lower frequency may be secondary to the ethnic and genetic differences, good access to medications, and compliance with clinical follow-up, and the hospital admission criteria in Canada [7].

The study findings for the common presentations of lupus flare (34.6% MSK symptoms, 25.5% renal manifestations, 24.5% skin rash, 16.3% serositis) differed from findings in other studies and other countries. Somaily et al. identified lupus nephritis as the most common lupus flare manifestation in Aseer, Saudi Arabia [8]. Lee et al. reported renal manifestations as the most common lupus flare presentation in the Canadian population [7]. Other most common manifestations of lupus flare among hospitalized patients include constitutional symptoms, hematologic disorders, and MSK symptoms [14-16]. Among patients admitted with infection, community-acquired pneumonia was the most common type of infection in this study, which is similar to the SLE patients admitted with infection in Catalonia, China, and Israel [13-15].

Only 2% of the SLE patients in the current study were admitted because of thromboembolic events, which is consistent with a study by Jallouli et al. in Tunisia (1.5%) and a study by Lee et al. in Canada (1.9%) [7,17]. Most SLE patients with deep venous thrombosis and minor pulmonary embolism do not need admission and instead can be treated as outpatients, which explains the lower rate of hospitalization among this group.

The low rate of adverse drug reactions (2%) among hospitalized patients in this study contrasts with other reports that indicated higher rates, between 5% and 8.1% [7,15]. The lower rate in the current study may be secondary to poor reporting of the adverse drug reactions for patients at the medical center as well as a lack of patient adherence to treatment and a lack of outpatient follow-up. The present study also demonstrated a lower rate of admission for pregnancy and labor complications (2%), compared with other reports (9-18.9%) [7,15], which may be explained by different patient characteristics at the authors’ center because it does not have a specialized obstetric ward.

The average length of hospitalization was higher in this study (mean 13.16 days) compared with other reports (mean 8.5 and 9.6 days) [5,7], which may be explained by the higher morbidity among patients at the authors’ center. In addition, the delay in performing some investigations, such as kidney biopsy, brain MRI, 24-hour urine collection for protein, could contribute to the prolonged hospitalization time.

Patients admitted with lupus flare in this study had a lower mean age compared with patients admitted with infection, which is consistent with the Catalonian study [14]. Most of the patients admitted in both groups were female, which is comparable to other reports [7,14,15,18,19]. No significant difference in the mean duration of hospitalization was noted between the two groups, similar to the observations reported by Lee et al. among the Canadian population [7]. No statistically significant difference in the laboratory results between the two groups was noted, except for a lower lymphocyte count of patients admitted with infection versus disease flare (Table 6). Lymphopenia is a well-known risk factor for infection in SLE patients [20].

The higher rate of prednisolone intake among patients admitted with lupus flare versus infection differs from previous reports, such as by Bosch et al., which indicated that the use of steroids is a risk factor for infection among SLE patients [21]. The higher rate of steroid use among patients admitted with lupus flare in the present study may be secondary to the disease severity and difficulty to control the disease manifestations among this group.

Most of the ICU patients had disease flare versus infection, which was similar to observations by Alvarez Barreneche et al. [22]. Other studies have also indicated infection as the main cause of ICU admission among SLE patients [23,24].

## Conclusions

Study results showed the two most common reasons for hospitalization among SLE patients were lupus flare and infection. Most of the patients admitted with lupus flare presented with MSK symptoms and renal manifestations. Among patients admitted with infection, the most common infection was community-acquired pneumonia. Patients with lupus flare were more likely to be taking steroids, whereas patients admitted with infection were more likely to have lymphopenia. Insuring patients’ compliance could decrease the frequency of hospitalization with lupus flare. In addition, ensuring updated vaccination may decrease the risk of hospitalization with infection. These measures altogether can lower the rate of hospitalization and decrease the morbidity and mortality of SLE patients.
